# Supplementary material for: COZOID: contact zone identifier for visual analysis of protein-protein interactions
Source: BMC Bioinformatics. 2018 Apr 6;19:125. doi: 10.1186/s12859-018-2113-6 (PMC5889581; doi:10.1186/s12859-018-2113-6)
Supplement: Supplementary file 2 — Software build. Executable binary file of the software tool. (ZIP 118,784 kb) [file 12859_2018_2113_MOESM2_ESM.zip › cozoid/java/docs/beansbinding-1.2.1-doc.zip!/index-files/index-16.html]

P-Index (Beans Binding)


---


|  |  |  |
| --- | --- | --- |
| **Overview** \| Package \| Class \| Use \| **Tree** \| **Deprecated** \| **Index** \| **Help** | |  |
| **PREV LETTER**   **NEXT LETTER** | **FRAMES**    **NO FRAMES**     **All Classes** |  |


A B C D E F G H I J K L M N O P Q R S T U V W 

---


## **P**

**paramString()** - Method in class org.jdesktop.beansbinding.AutoBinding: Returns a string representing the internal state of the `Binding`. **paramString()** - Method in class org.jdesktop.beansbinding.Binding: Returns a string representing the internal state of the `Binding`. **paramString(Class[])** - Static method in class org.jdesktop.el.impl.util.ReflectionUtil: **parent** - Variable in class org.jdesktop.el.impl.parser.SimpleNode: **parse(String)** - Static method in class org.jdesktop.el.impl.parser.ELParser: **ParseException** - Exception in org.jdesktop.el.impl.parser: This exception is thrown when parse errors are encountered. **ParseException(Token, int[][], String[])** - Constructor for exception org.jdesktop.el.impl.parser.ParseException: This constructor is used by the method "generateParseException" in the generated parser. **ParseException()** - Constructor for exception org.jdesktop.el.impl.parser.ParseException: The following constructors are for use by you for whatever purpose you can think of. **ParseException(String)** - Constructor for exception org.jdesktop.el.impl.parser.ParseException: **PLUS** - Static variable in interface org.jdesktop.el.impl.parser.ELParserConstants: **prefix** - Variable in class org.jdesktop.el.impl.lang.FunctionMapperImpl.Function: **prefix** - Variable in class org.jdesktop.el.impl.parser.AstFunction: **prevCharIsCR** - Variable in class org.jdesktop.el.impl.parser.SimpleCharStream: **prevCharIsLF** - Variable in class org.jdesktop.el.impl.parser.SimpleCharStream: **PRIMITIVE\_NAMES** - Static variable in class org.jdesktop.el.impl.util.ReflectionUtil: **PRIMITIVES** - Static variable in class org.jdesktop.el.impl.util.ReflectionUtil: **Property**<S,V> - Class in org.jdesktop.beansbinding: `Property` defines a uniform way to access the value of a property. **Property()** - Constructor for class org.jdesktop.beansbinding.Property: **property** - Variable in class org.jdesktop.el.impl.parser.AstValue.Target: **property** - Variable in class org.jdesktop.swingbinding.adapters.BeanAdapterBase: **PropertyHelper**<S,V> - Class in org.jdesktop.beansbinding: An abstract subclass of `Property` that helps with the management of `PropertyStateListeners` by implementing the methods for adding, removing, and getting listeners. **PropertyHelper()** - Constructor for class org.jdesktop.beansbinding.PropertyHelper: Create a `PropertyHelper` that manages listeners for multiple source objects. **PropertyHelper(boolean)** - Constructor for class org.jdesktop.beansbinding.PropertyHelper: Create a `PropertyHelper`, specifying whether it manages listeners for multiple source objects, or ignores the source object argument when dealing with listeners **PropertyNotFoundException** - Exception in org.jdesktop.el: Thrown when a property could not be found while evaluating a `ValueExpression` or `MethodExpression`. **PropertyNotFoundException()** - Constructor for exception org.jdesktop.el.PropertyNotFoundException: Creates a `PropertyNotFoundException` with no detail message. **PropertyNotFoundException(String)** - Constructor for exception org.jdesktop.el.PropertyNotFoundException: Creates a `PropertyNotFoundException` with the provided detail message. **PropertyNotFoundException(Throwable)** - Constructor for exception org.jdesktop.el.PropertyNotFoundException: Creates a `PropertyNotFoundException` with the given root cause. **PropertyNotFoundException(String, Throwable)** - Constructor for exception org.jdesktop.el.PropertyNotFoundException: Creates a `PropertyNotFoundException` with the given detail message and root cause. **PropertyNotWritableException** - Exception in org.jdesktop.el: Thrown when a property could not be written to while setting the value on a `ValueExpression`. **PropertyNotWritableException()** - Constructor for exception org.jdesktop.el.PropertyNotWritableException: Creates a `PropertyNotWritableException` with no detail message. **PropertyNotWritableException(String)** - Constructor for exception org.jdesktop.el.PropertyNotWritableException: Creates a `PropertyNotWritableException` with the provided detail message. **PropertyNotWritableException(Throwable)** - Constructor for exception org.jdesktop.el.PropertyNotWritableException: Creates a `PropertyNotWritableException` with the given root cause. **PropertyNotWritableException(String, Throwable)** - Constructor for exception org.jdesktop.el.PropertyNotWritableException: Creates a `PropertyNotWritableException` with the given detail message and root cause. **PropertyResolutionException** - Exception in org.jdesktop.beansbinding: `PropertyResolutionExceptions` can be thrown at various points in the life cycle of a `Property`. **PropertyResolutionException(String)** - Constructor for exception org.jdesktop.beansbinding.PropertyResolutionException: Creates a `PropertyResolutionException` with the given message. **PropertyResolutionException(String, Exception)** - Constructor for exception org.jdesktop.beansbinding.PropertyResolutionException: Creates a `PropertyResolutionException` with the given message and cause. **propertyStateChanged(PropertyStateEvent)** - Method in interface org.jdesktop.beansbinding.PropertyStateListener: Called to notify the listener that a change of state has occurred to one of the `Property` instances upon which the listener is registered. **PropertyStateEvent** - Class in org.jdesktop.beansbinding: An event characterizing a change in a `Property's` state for a particular source object. **PropertyStateEvent(Property, Object, boolean, Object, Object, boolean, boolean)** - Constructor for class org.jdesktop.beansbinding.PropertyStateEvent: Creates an instance of `PropertyStateEvent` characterizing a change in a `Property's` state for a particular source object. **PropertyStateListener** - Interface in org.jdesktop.beansbinding: `PropertyStateListeners` are registerd on `Property` instances, to be notified when the state of the property changes. **providesAdapter(Class<?>, String)** - Method in interface org.jdesktop.beansbinding.ext.BeanAdapterProvider: **providesAdapter(Class<?>, String)** - Method in class org.jdesktop.swingbinding.adapters.AbstractButtonAdapterProvider: **providesAdapter(Class<?>, String)** - Method in class org.jdesktop.swingbinding.adapters.JComboBoxAdapterProvider: **providesAdapter(Class<?>, String)** - Method in class org.jdesktop.swingbinding.adapters.JListAdapterProvider: **providesAdapter(Class<?>, String)** - Method in class org.jdesktop.swingbinding.adapters.JSliderAdapterProvider: **providesAdapter(Class<?>, String)** - Method in class org.jdesktop.swingbinding.adapters.JSpinnerAdapterProvider: **providesAdapter(Class<?>, String)** - Method in class org.jdesktop.swingbinding.adapters.JTableAdapterProvider: **providesAdapter(Class<?>, String)** - Method in class org.jdesktop.swingbinding.adapters.JTextComponentAdapterProvider: **putContext(Class, Object)** - Method in class org.jdesktop.el.ELContext: Associates a context object with this `ELContext`. **putContext(Class, Object)** - Method in class org.jdesktop.el.impl.lang.EvaluationContext

---


|  |  |  |
| --- | --- | --- |
| **Overview** \| Package \| Class \| Use \| **Tree** \| **Deprecated** \| **Index** \| **Help** | |  |
| **PREV LETTER**   **NEXT LETTER** | **FRAMES**    **NO FRAMES**     **All Classes** |  |


A B C D E F G H I J K L M N O P Q R S T U V W 

---
